# Supplementary material for: fMRI neurofeedback in the motor system elicits bidirectional changes in activity and in white matter structure in the adult human brain
Source: Cell Rep. 2021 Oct 26;37(4):109890. doi: 10.1016/j.celrep.2021.109890 (PMC8961413; doi:10.1016/j.celrep.2021.109890)
Supplement: Document S1. Figures S1–S3 and Tables S1–S3 [file mmc1.pdf]

Cell Reports, Volume *37*

Supplemental information

fMRI neurofeedback in the motor system elicits bidirectional changes in activity and in white matter structure in the adult human brain

Cassandra Sampaio-Baptista, Heather F. Neyedli, Zeena-Britt Sanders, Kata Diosi, David Havard, YunYing Huang, Jesper L.R. Andersson, Michael Lühr, Rainer Goebel, and Heidi Johansen-Berg

Supplementary Figures and Results

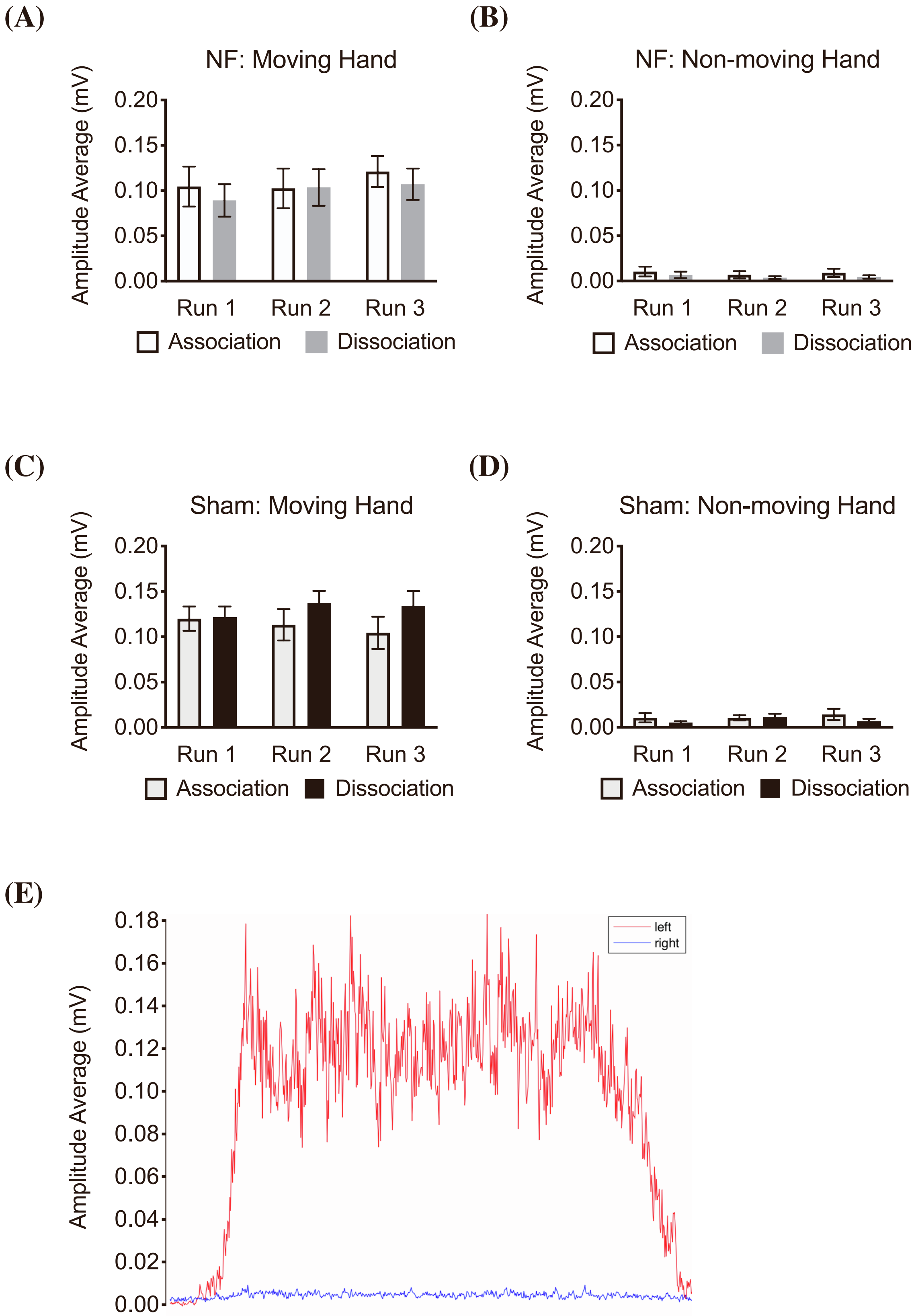

Supplementary Figure 1. EMG results. Related to Figure 2. Mixed Design ANOVA that included group, condition, run and hand as factors revealed an effect of hand, with the average amplitude EMG of the moving (left) hand significantly higher than the non-moving

(right) hand (Fig S1A, B) ($F_{(1,14)} = 182.472$, $p < 0.001$). There was no effect of group ($F_{(1,14)} = 0.522$, $p = 0.482$), condition ($F_{(1,14)} = 0.006$, $p = 0.938$), or run (($F_{(2,28)} = 1.508$, $p = 0.239$) nor any interaction effects (all $p > 0.05$). (A) Average amplitude (mV) of the left hand for the NF group. (B) Average amplitude of the right hand for the NF group. (C) Average amplitude (mV) of the left hand for the Sham group. (D) Average amplitude of the right hand for the Sham group (E) Example from one participant of the average activation for each hand. Error bars represent SEM.

Questionnaire
A. How much control over the blue bar did you feel you had? (1-not in control, 5-full in control)
B. Which strategies did you use? rate on a scale of 1 to 5 how successful the strategy was (1 – unsuccessful strategy; 5 – successful strategy):
1. Focusing more on the moving hand
2. Focusing less on the non-moving hand
3. Physically relaxing the non-moving hand
4. Increasing the rate of movement
5. Increasing the force of the movement
6. Increasing the size of the movement
7. Tapping the fingers in a fixed sequence
8. Tapping the fingers in a random sequence
9. Opening and closing the hand
10. Making grasping movements
11. Imagining bilateral movements (while keeping the right hand still)
12. - Other strategies (score)

Supplementary Table 1. Debriefing Questionnaire. Related to Figure 2. Participants filled in a questionnaire after the neurofeedback session.

Questionnaire								
A. How much control over the blue bar did you feel you had? (1-not in control, 5-full in control)	NF Group				Sham Group			
	Association		Dissociation		Association		Dissociation	
	Mean	SD	Mean	SD	Mean	SD	Mean	SD
	2.63	1.2	3.1	0.98	2.70	0.59	2.95	0.64

Supplementary Table 2. Debriefing Questionnaire Results. Related to Figure 2. There were no significant differences between sham and NF groups in response to the question "how much control over the blue bar did you feel you had?" for the Association condition (Mann-Whitney U test; $Z = 0.327$, $p=0.756$) or the Dissociation condition (Mann-Whitney U test; $Z = -1.081$, $p=0.314$). Within group, there were no significant differences in response to this question between experimental conditions (Wilcoxon signed-rank test; real NF group: $Z = -1.294$, $p=0.196$; Sham group: $Z = -0.991$, $p=0.322$). With regards to participants' rankings on how useful they found the strategies they tried (Supplementary Table 1, Question B), there was no difference in the rankings between groups for the Association condition (Mann-Whitney U test; $Z = 0.986$, $p = 0.349$) or the Dissociation condition (Mann-Whitney U test; $Z = 0.458$, $p = 0.654$). Overall, participants of both groups perceived similar degrees of control and considered that the strategies were similarly successful.

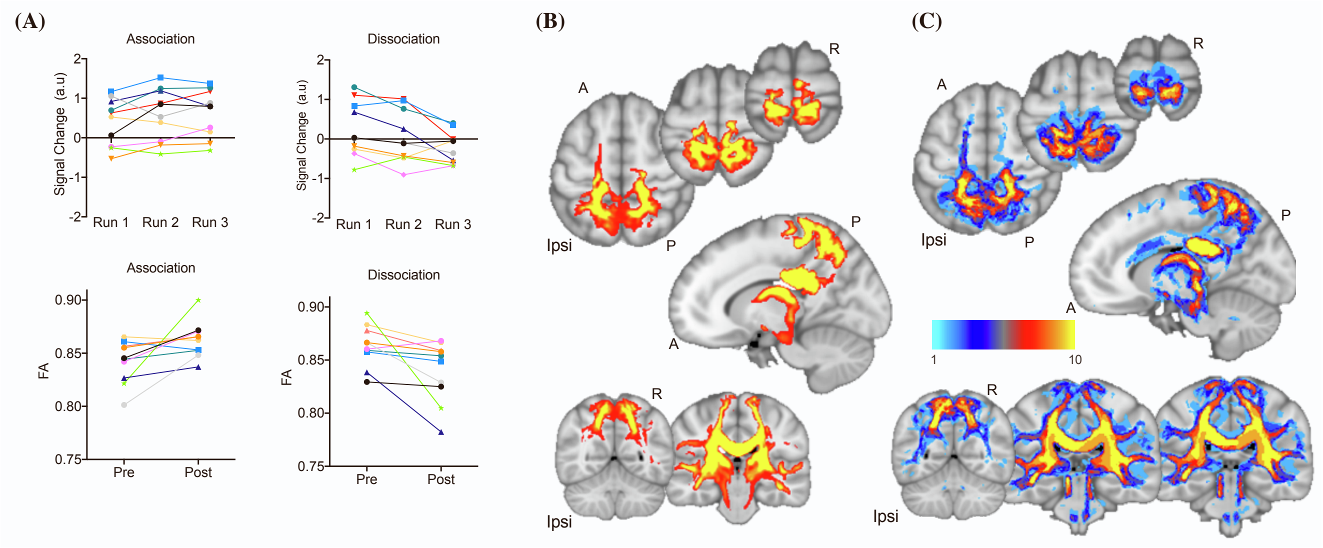

Supplementary Figure 2. Related to Figure 2 and 3. Individual data and tractography results (A) Plots showing colour-coded individual data over time for the Real NF group. Top: Ipsilateral S1M1 activity during fMRI NF for each condition over the 3 runs. Bottom: Pre and Post average FA values for each condition of the significant cluster represented in Figure 3A of the main manuscript. (B-C) The significant FA cluster connects to sensorimotor and posterior parietal areas. (B) Mean connectivity map (red-yellow) of all participants of the FA cluster. Yellow areas depict higher connectivity probability (threshold > 100). (C) Population connectivity map showing the overlap between participants (Light blue represents 1 participant – Yellow represents tracts common to the 10 participants). Tractography map is overlaid on the MNI template. Ipsi – Ipsilateral Hemisphere, A- Anterior, P- Posterior, R - Right.

Participant	Session	Condition
1	1	Association
2	2	Dissociation
3	2	Dissociation
4	1	Dissociation
5	1	Association
6	2	Association
7	1	Dissociation
8	2	Dissociation
9	1	Dissociation
10	2	Association

Supplementary Table 3. Related to Figure 3. Session and condition of best performance for each participant. We expected that changes in white matter structure would reflect successful modulation of activity with NF. For each participant we therefore identified which of the two NF conditions they performed best and computed correlations (see Supplementary Figure 3. A and B).

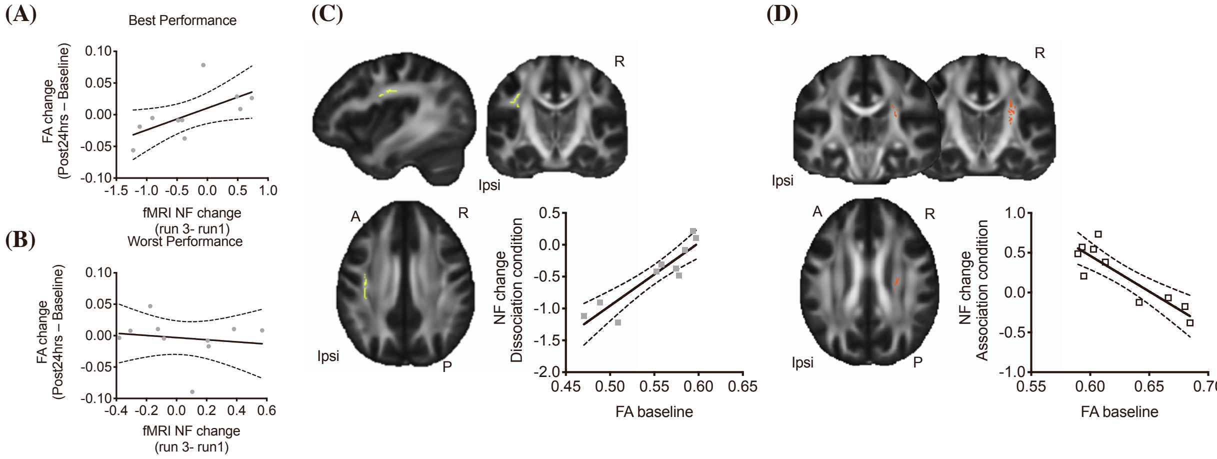

Supplementary Figure 3. Related to Figure 3. Preliminary findings: Correlations between Neurofeedback performance and changes in white matter structure and Baseline FA correlates with NF performance. (A) Significant correlation between the fMRI activity change (run 3 – run 1) and change in FA following the training session with the best performance (Post24hrs – Baseline) (n=10, Spearman's Rho = 0.72, p = 0.02, 2-tail; Supplementary Figure 3A). (B) No such correlation was found for the worse NF session (n=10, Spearman's Rho = - 0.16, p = 0.66, 2-tail; Supplementary Figure 3B). These correlations were significantly different from each other (test of the difference between two dependent correlations; z = 2.058, p = 0.039, 2-tail. Due to the small sample these results should be considered preliminary findings but suggest that following effective NF training, changes in structure are potentially related to how effectively the participant modulated iS1M1 activity. (C) Within the real NF group we tested whether baseline measures of FA correlated with change in fMRI (run 3 – run 1) for each condition (n=10). Non-parametric permutations voxel-wise analysis of the whole skeleton revealed a significant positive correlation ($p < 0.05$, corrected) between baseline FA and NF fMRI change for the Dissociation condition in the ipsilateral (left) superior longitudinal fasciculus (SLF) (represented in yellow). (D) A trend towards a significant negative correlation (p = 0.06, corrected) (represented in red) between baseline FA and NF change was found in the contralateral (right) corticospinal tract for the Association condition. Plots in (C) and (D) are shown for visualization of range of values and not for inference. Significant clusters are superimposed on the FMRIB FA template. Ipsi – Ipsilateral Hemisphere, A-Anterior, P-Posterior, R – Right. Dotted lines represent SEM.
