# Supplementary material for: fMRI neurofeedback in the motor system elicits bidirectional changes in activity and in white matter structure in the adult human brain
Source: Cell Rep. 2021 Oct 26;37(4):109890. doi: 10.1016/j.celrep.2021.109890 (PMC8961413; doi:10.1016/j.celrep.2021.109890)
Supplement: Document S2. Article plus supplemental information [file mmc2.pdf]

Cell Reports

Report

fMRI neurofeedback in the motor system elicits bidirectional changes in activity and in white matter structure in the adult human brain

Graphical abstract

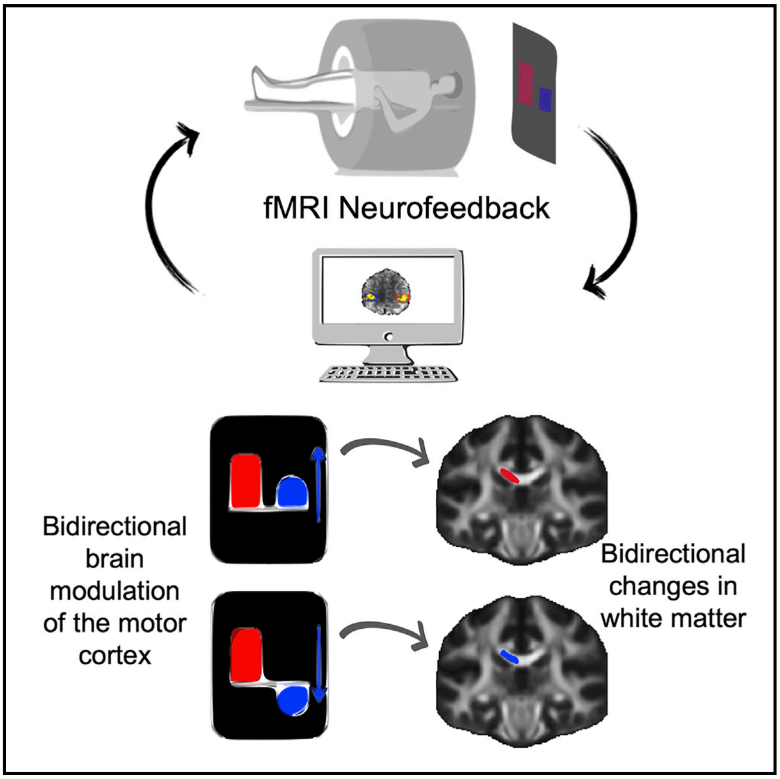

Highlights

- fMRI neurofeedback is used to bidirectionally modulate sensorimotor activity
- Brain-activity self-regulation results in bidirectional white matter alterations
- White matter structural changes relate to the direction of the activity regulation
- Neurofeedback can be used to endogenously and directionally alter brain structure

Authors

Cassandra Sampaio-Baptista, Heather F. Neyedli, Zeena-Britt Sanders, ..., Michael Lühr, Rainer Goebel, Heidi Johansen-Berg

Correspondence

cassandra.sampaiobaptista@glasgow.ac.uk

In brief

Up- and downregulation of neuronal activity alter myelin and white matter in animal models. Sampaio-Baptista et al. demonstrate using fMRI neurofeedback that bidirectional regulation of sensorimotor activity results in bidirectional changes in white matter in adult humans. fMRI neurofeedback can be used to directionally modulate brain structure.

Sampaio-Baptista et al., 2021, Cell Reports *37*, 109890
October 26, 2021 © 2021 The Authors.
https://doi.org/10.1016/j.celrep.2021.109890

CellPress

Cell Reports

CellPress
OPEN ACCESS

Report

fMRI neurofeedback in the motor system elicits bidirectional changes in activity and in white matter structure in the adult human brain

Cassandra Sampaio-Baptista,[1,2,5,*] Heather F. Neyedli,[1,3] Zeena-Britt Sanders,[1] Kata Diosi,[1] David Havard,[1] YunYing Huang,[1] Jesper L.R. Andersson,[1] Michael Lühr,[4] Rainer Goebel,[4] and Heidi Johansen-Berg[1]

[1]Wellcome Centre for Integrative Neuroimaging, FMRIB, Nuffield Department of Clinical Neurosciences, University of Oxford, John Radcliffe Hospital, Headington, Oxford OX3 9DU, UK

[2]Institute of Neuroscience and Psychology, College of Medical, Veterinary and Life Sciences, University of Glasgow, 62 Hillhead Street, Glasgow G12 8QB, UK

[3]Department of Kinesiology, School of Health and Human Performance, Dalhousie University, 6230 South Street, Halifax, NS B3H 4R2, Canada

[4]Department of Cognitive Neuroscience, Maastricht University, Oxfordlaan 55, 6229 Maastricht, the Netherlands

[5]Lead contact

*Correspondence: cassandra.sampaiobaptista@glasgow.ac.uk

https://doi.org/10.1016/j.celrep.2021.109890

SUMMARY

White matter (WM) plasticity supports skill learning and memory. Up- and downregulation of brain activity in animal models lead to WM alterations. But can bidirectional brain-activity manipulation change WM structure in the adult human brain? We employ fMRI neurofeedback to endogenously and directionally modulate activity in the sensorimotor cortices. Diffusion tensor imaging is acquired before and after two separate conditions, involving regulating sensorimotor activity either up or down using real or sham neurofeedback (n = 20 participants × 4 scans). We report rapid opposing changes in corpus callosum microstructure that depend on the direction of activity modulation. Our findings show that fMRI neurofeedback can be used to endogenously and directionally alter not only brain-activity patterns but also WM pathways connecting the targeted brain areas. The level of associated brain activity in connected areas is therefore a possible mediator of previously described learning-related changes in WM.

INTRODUCTION

Behavioral training changes the structure of white matter pathways (Sampaio-Baptista and Johansen-Berg, 2017). These can be detected at short and long timescales with diffusion tensor imaging (DTI) (Hofstetter et al., 2013; Scholz et al., 2009; Taubert et al., 2010). While DTI-derived measures, such as fractional anisotropy (FA), are nonspecific and modulated by a variety of white matter features, alterations in white matter associated with learning have been partially related to myelin increases in rodents (Sampaio-Baptista et al., 2013, 2020). Myelination can be bidirectionally altered by neuronal activity (Baraban et al., 2018; Demerens et al., 1996; Gibson et al., 2014; Hines et al., 2015; Mitew et al., 2018; Stevens et al., 1998). Importantly, new oligodendrocytes and myelin, formed during adulthood, play an essential role in motor skill acquisition (McKenzie et al., 2014; Xiao et al., 2016) and memory (Pan et al., 2020; Steadman et al., 2020). This suggests that learning is supported not only by neuronal changes, such as synaptic plasticity, but also by adjunct changes in myelination (Long and Corfas, 2014). Recent animal studies indicate that axon caliber and nodes of Ranvier can also be regulated by neuronal activity (Arancibia-Cárcamo et al., 2017; Ford et al., 2015; Sinclair et al., 2017). Therefore, a number of white matter components are regulated by activity-dependent mechanisms. However, it has not yet been tested in humans whether bidirectional brain-activity manipulation can result in white matter changes. Behavioral training is associated with widespread brain activity, making it difficult to test specific predictions about the location and direction of white matter changes (Thomas and Baker, 2013). In contrast to behavioral training, direct modulation of activity within specific brain regions might be predicted to evoke directionally dependent changes in white matter structure. For example, 30 min of optogenetic stimulation of premotor neurons led to rapid increases in oligodendrocyte precursor cells (OPCs) proliferation and differentiation within 24 h (Gibson et al., 2014). A powerful approach to test these effects in humans is to employ functional magnetic resonance imaging (fMRI) neurofeedback (NF) to modulate focal activity in combination with DTI measures.

fMRI NF is a closed-loop technique that allows participants to modulate their own brain activity by measuring and analyzing it in almost real time. NF is currently being investigated as a potential tool to alter abnormal activity patterns in variety of clinical conditions (Sitaram et al., 2017; Wang et al., 2018). fMRI NF allows

© 2021 The Authors.
This is an open access article under the CC BY license (http://creativecommons.org/licenses/by/4.0/).

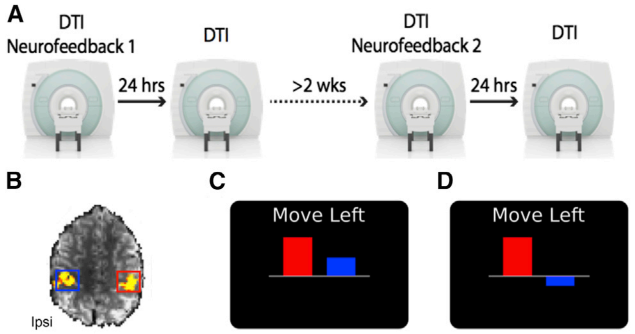

Figure 1. Timeline and neurofeedback display

(A) Participants in the real NF group and sham group experienced two different NF conditions, in a counterbalanced design, at least 2 weeks apart, with DTI acquired before each NF session and again 24 h later.

(B) Functional localizer of an example participant. S1M1 regions of interest were identified by asking the participants to move their right or left fingers sequentially. Ipsi, ipsilateral.

(C) Example NF display for the association condition.

(D) Example NF display for the dissociation condition.

for spatially specific alteration of brain-activity patterns, with studies showing encouraging behavioral or physiological effects (Neyedli et al., 2018; Ramot et al., 2016; Shibata et al., 2011; Sitaram et al., 2017; Young et al., 2017). So far, most NF studies have focused on functional effects, and only two, including a EEG-NF study, have assessed NF effects on the structure of long-range connections (Ghaziri et al., 2013; Marins et al., 2019). However, despite promising findings, EEG has poor spatial resolution, and although motor imagery can be a useful paradigm in neurofeedback, it elicits widespread activity in the brain. Therefore, these studies (Ghaziri et al., 2013; Marins et al., 2019) cannot conclusively establish direct relationships between specific brain-activity modulation and specific structural changes. Further, neither tested the effects of bidirectional modulation on white matter structure.

To overcome these limitations and establish direct links between specific brain-activity modulation through self-regulation and directional structural changes, we tested whether increasing and decreasing sensorimotor activity has differential effects on white matter structure in the same participants. Specifically, we used real-time fMRI NF at 7 Tesla to manipulate the activity of the sensorimotor cortices (S1M1s) in opposite directions in the same participants and tested for effects on white matter structure against a sham group (n = 10 participants per group × 4 scans; 22–38 years old, 15 female). We focused on executed movements to increase the specificity of the modulated regions. This allowed us to also address the degree to which activity in ipsilateral motor cortex can be bidirectionally modified with fMRI NF in healthy individuals during executed movements, which is relevant to NF applications to motor neurorehabilitation contexts.

RESULTS

We employed a mixed-design approach with both within-subject factors (condition and time) and between-subject factors (real and sham). Each participant was scanned 4 times and experienced two different NF conditions (only one NF condition was experienced in each session), with DTI acquired before each condition and again 24 h later (Figure 1A; see also STAR Methods). The order of the conditions was counterbalanced, and to minimize possible carry-over effects after a single NF session the two conditions were spaced more than 2 weeks apart (see STAR Methods). During NF, participants were instructed to modulate the height of two bars (representing activity in ipsilateral and contralateral S1M1) on a visual display, by moving *only* their left hand during 30 s movement blocks, which alternated with 30 s rest blocks. In the “association condition,” participants were required to co-activate both S1M1s (Figures 1B and 1C), while in the “dissociation condition” they were required to maximize contralateral S1M1 activity, while minimizing ipsilateral S1M1 activity (Figures 1B and 1D). Participants in the sham group received the same instructions but were shown the NF videos of a matched participant in the real NF group and experienced the same two conditions (association and dissociation). 80 scans were successfully completed, 28 participants were enrolled, and complete datasets were obtained in 20 participants. For each NF condition, participants trained for approximately 20 min (in 3 or 4 runs of ~6 min). EMG was used to monitor hand movements online and as expected the muscle activity of the moving (left) hand was significantly higher than the non-moving (right) hand and was similar between real NF and sham groups (Figure S1). A debriefing questionnaire (Table S1) revealed that both groups felt in control of the NF (Table S2).

We assessed fMRI activity to test whether participants could modulate S1M1 activity with feedback as instructed. We first analyzed a signal change within the regions selected during NF using a mixed-design ANOVA including within-subject factors of condition (association, dissociation) and time (run 1, 2, 3) and between-subject factor of group (real, sham). Instructions required participants to increase ipsilateral S1M1 (iS1M1) activity for the association condition and decrease it for the dissociation condition. Compared to the sham group, participants in the real NF group were able to modulate activity in iS1M1 as instructed (Figures 2A and 2B; main effect of condition: $F_{(1,18)} = 8.53$, $p = 0.009$; condition × group interaction: $F_{(1,18)} = 12.082$, $p = 0.003$; condition × time × group interaction $F_{(2,36)} = 5.03$, $p = 0.012$). By contrast, no effects of (or interactions with) group were found for the contralateral S1M1 region of interest (ROI), with both groups strongly activating this ROI for both conditions (Figures 2D and 2E).

Following these results, a repeated-measures ANOVA was used to explore effects on the iS1M1 within the real NF group (condition [association, dissociation] and time (run 1, 2, 3) were used as factors). Participants within the real NF group were able to modulate iS1M1 activity in opposite directions, with greater, and increasing, activity in the association condition compared to the dissociation condition (Figure 2A: main effect of condition: $F_{(1,9)} = 32.045$, $p = 0.00031$; condition × time interaction: $F_{(2,18)} = 6.665$, $p = 0.007$; see also Figure S2A). Voxel-wise analysis within the real NF group revealed specific clusters of significantly greater activity in motor areas, including the ipsilateral hand knob, in the association compared to the dissociation condition (Figure 2C). No other significant clusters were found.

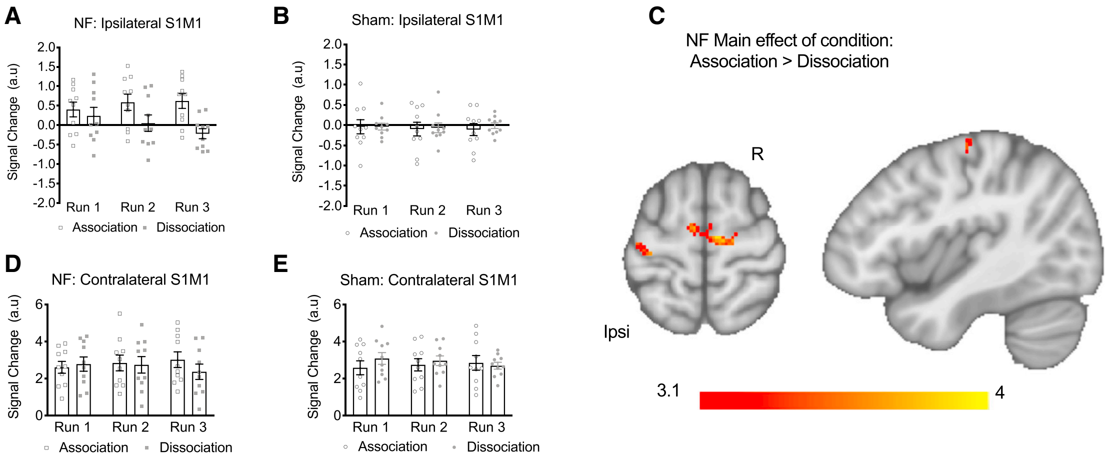

Figure 2. Participants were able to modulate iS1M1activity with real NF

(A) Participants in the real NF group had lower iS1M1 activity in the dissociation condition compared to the association condition (n = 10 per group).

(B) iS1M1 activity of the sham group over the 3 runs.

(C) Voxel-wise analysis showing significantly higher activity in the association condition compared to the dissociation condition in iS1M1 ($p < 0.05$, corrected) in the real NF group.

(D and E) Instructions required participants to increase contralateral S1M1 (cS1M1) activity for both conditions and both groups. Results showed no main effects or interactions with group, and no main effects of time or condition on signal change within the cS1M1 ROI. There was a significant interaction effect of condition × time, which was further explored ($F_{(2,36)} = 6.25$, $p = 0.005$). This was driven by an effect of time for the dissociation condition ($F_{(2,36)} = 5.309$, $p = 0.01$), showing both groups decrease activity over time within this condition. No effects of time were identified for the association condition ($F_{(2,36)} = 2.115$, $p = 0.135$). (D) Real NF group contralateral activity in the S1M1 ROI over the 3 runs. (E) Sham-group contralateral activity in the S1M1 ROI over the 3 runs. A.u., arbitrary units; Ipsi, ipsilateral hemisphere; R, right. Error bars represent SEM.

No significant clusters were found in the sham group for the same comparison, showing that sham participants did not differentially modulate their brain activity between conditions. The fMRI results therefore demonstrate that the two real NF conditions differ in iS1M1 activity, with greater activity seen in the association condition compared to the dissociation condition.

We went on to test whether this activity modulation resulted in alterations in white matter, as measured by FA, an indirect measure of white matter microstructure previously shown to be sensitive to learning-related white matter plasticity (Sampaio-Baptista et al., 2013; Scholz et al., 2009). Voxel-wise FA maps were calculated from DTI scans acquired before and 24 h after each NF condition. To accommodate the mixed design nature of this study, FA change maps (post-pre for each condition) were first calculated for each condition and each group. Then maps of the difference in FA change between conditions (dissociation condition FA change – association condition FA change = condition difference) were calculated for each subject. The resulting maps were then compared between groups.

We used a data-driven approach and performed whole-skeleton voxel-wise non-parametric permutation testing of these between-group differences. This revealed a statistically significant cluster in the corpus callosum, and no other clusters were identified elsewhere in the brain (Figure 3A) ($p < 0.05$, corrected). This reflected a positive FA change for the association condition and a negative FA change for the dissociation condition within the real NF group (Figure 3B; Figure S2A) compared to the sham group. Tractography from this cluster identified pathways connecting sensorimotor and parietal cortices (Figures S2B and S2C). See supplemental information for exploratory analysis of relationships between changes in white matter and successful modulation of activity with real NF (Table S3; Figures 3A and 3B) and relationships between baseline white matter structure and NF performance (Figures S3C and S3D).

DISCUSSION

The results support the hypothesis that bidirectional activity modulation of ipsilateral sensorimotor activity during executed hand movement can be achieved via NF and that this results in rapid, directional, and localized changes in white matter structure.

Likely more than one cellular mechanism underlies the structural findings. FA is modulated by several white matter features such as myelination, axon density, and caliber and potentially by astrocyte morphology, cell swelling, or changes in membrane fluidity (Sampaio-Baptista and Johansen-Berg, 2017). Myelination is an attractive potential mechanism because neuronal activity can bidirectionally regulate myelin formation and compaction and OPCs proliferation and differentiation (Demerens et al., 1996; Gibson et al., 2014; Stevens et al., 1998), and such effects occur over similar timescales to the ones used here (Xiao et al., 2016). For example, 30 min of optogenetic stimulation of premotor neurons led to rapid increases in oligodendrocyte precursor cells (OPCs) proliferation and differentiation within 24 h (Gibson et al., 2014). Furthermore, recently matured oligodendrocytes can form, extend, and retract myelin segments within 24 h (Czopka et al., 2013; Watkins et al., 2008). Preexisting oligodendrocytes can also potentially contribute to myelin remodeling (Dutta et al., 2018; Mitew et al., 2018; Yeung et al., 2014). This

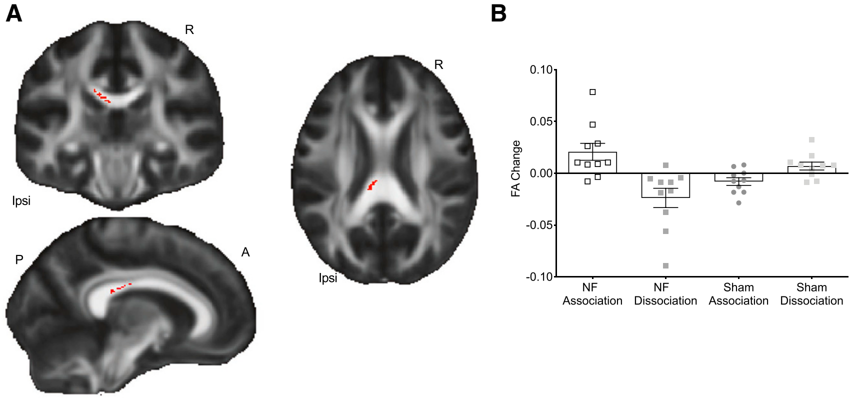

Figure 3. NF training resulted in changes in white matter FA in the corpus callosum

(A) Significant FA cluster (in red) of the between-group contrast (n = 10 per group, p = 0.05, corrected).

(B) The plot represents the individual participant mean FA change values within the significant cluster represented in the FA map and is shown for visualization of range of values and effect direction and not for inference.

has been particularly demonstrated following injury in the cortex (Bacmeister et al., 2020; Duncan et al., 2018). However, strong evidence for this process in the healthy intact brain is currently scarce, and the timescale at which this occurs is unknown. Concurrently, other structural properties such as axon density and caliber, astrocyte morphology, cell swelling, or membrane fluidity are also altered in response to neuronal activity and could also underlie the detected DTI effects (Blumenfeld-Katzir et al., 2011; Sampaio-Baptista and Johansen-Berg, 2017; Sinclair et al., 2017).

The structural findings suggest that alterations in the elicited brain activity is a possible mediator of previously described experience-related white matter changes in the human brain resulting from behavioral interventions (Hofstetter et al., 2013; Scholz et al., 2009). However, given the complex nature of the BOLD signal it is not straightforward to attribute BOLD fMRI increases or decreases to either net excitation or net inhibition (Devor et al., 2007; Logothetis, 2008), though some cellular specific mechanisms that contribute to BOLD have been recently described (Uhlirova et al., 2016).

The anatomical site of the detected FA changes signals that successful modulation of left sensorimotor activity by performing left-hand movements resulted in changes mainly in the fibers that connect to the opposite hemisphere. This indicates that modulation of ipsilateral activity occurred via callosal connections, resulting in structural alterations in the connections between the two cortices. The corpus callosum is a relatively coherent fiber bundle as such small changes might be easier to detect in this location, whereas white matter closer to the cortex contains more crossing fibers and so effects of structural modulation on DTI metrics could be harder to detect.

Activity in the ipsilateral S1M1 ROI showed clear modulation with real NF within a single session, which is faster than most previous motor NF studies. This discrepancy might be due to participants using real movements here as opposed to motor imagery, which has been more commonly used previously. A proportion of participants are unable to perform motor imagery, with or without NF (Auer et al., 2015; Chiew et al., 2012; Marins et al., 2019). Further, in our paradigm all participants are constantly being rewarded through continual updating of the red bar that represents their contralateral S1M1 activity, and this might motivate participants to engage in the task from the start.

Activity in the contralateral ROI remained fairly similar across conditions and groups, despite instructions to increase contralateral activity. Given that hand movements strongly elicit contralateral sensorimotor activity in healthy participants, it is likely that this is due to a ceiling effect, with gains in activity not easily achieved. By contrast, there is typically very little activity in ipsilateral S1M1 during this task in healthy individuals and so more scope for modulation. Future studies employing several training sessions should assess whether further training leads to progressive increases in contralateral activity, particularly in patient groups with motor deficits or in older populations who may have lower activity in this region at baseline.

One challenge for NF clinical application is the high degree of variability in response (Sitaram et al., 2017). Many studies identify "responders" and "non-responders" to NF, and the individual factors that determine NF success are not well understood. Recent work has started to explore factors that relate to variability in NF performance in order to identify predictors (Haugg et al., 2020). Here, we tested whether any baseline variables correlated with NF success. These preliminary findings indicate that higher ipsilateral SLF FA at baseline is associated with higher ipsilateral sensorimotor fMRI change. Additionally, higher contralateral corticospinal FA at baseline is associated with lower performance in increasing ipsilateral activity with NF. Both these results indicate that high baseline FA in motor-related pathways is associated with worse NF performance, implying that highly structurally connected motor networks might be harder to modulate via NF. Future studies should confirm and expand on these results. Still, these findings open the possibility of using structural imaging to predict NF performance, explain inter-individual variability, and potentially tailor the intervention to the needs of the participants. For instance, more NF sessions might be necessary to change the activity of a highly structurally connected network.

This study is relevant for NF applications in therapeutic contexts and in particular as an adjunct approach to motor neurorehabilitation. The two conditions tested here could potentially be used as alternative interventions in stroke patients. Following stroke, alterations in activity across the motor system are evident, with changes in activity in ipsilateral (contralesional) motor cortex a particular focus of interest (Johansen-Berg et al., 2002b). Whether this activity should be suppressed or amplified is a matter of debate and the optimal approach might well vary between patients. For instance, decreasing motor activity of

the ipsilateral (spared) hemisphere, while increasing it in the affected side, is a potential route for improving motor function after stroke, particularly in patients with low levels of impairment (Johansen-Berg, 2003, 2007; Johansen-Berg et al., 2002a). On the other hand, enhancing activity in the ipsilateral motor cortex may be beneficial for patients with more severe impairment (Bradnam et al., 2012; McDonnell and Stinear, 2017). Behavioral, motor evoked-responses (MEPs) (Stinear et al., 2007) and MRI markers (Sampaio-Baptista et al., 2018; Stinear and Ward, 2013) could be used as predictors for tailoring the NF intervention to the individual, including which brain areas to target and in which direction. This study shows that NF is a promising tool to manipulate sensorimotor brain patterns and to modulate intact white matter structure through this process, potentially leading to improved motor function in stroke. Whether these findings extend to cognitive systems, including memory or emotion, remains an open question, but white matter plasticity research suggests this property is not specific to the motor system (Hofstetter et al., 2013; Sampaio-Baptista and Johansen-Berg, 2017). In a wider context, these findings indicate fMRI NF can potentially be used to specifically and directionally modulate not only function but also white matter structure in a range of neurological and neuropsychiatric conditions.

This work has several limitations. While counterbalancing was used to correct for progressive errors and conditions were kept apart by more than 2 weeks, we cannot exclude the possibility of carryover effects. Due to the extensive scanning time employed in this study, we did not explore gray matter volumetric changes. For the same reasons, we were also unable to assess transfer effects by evaluating activity changes in the absence of NF. Due to the small sample size, replication and extension of these findings to other populations are essential steps. A larger sample size would also allow for further assessment of the relationship between NF response and structural changes, as well as potential identification of structural predictors.

STAR★METHODS

Detailed methods are provided in the online version of this paper and include the following:

- KEY RESOURCES TABLE
- RESOURCE AVAILABILITY
  - Lead contact
  - Materials availability
  - Data and code availability
- EXPERIMENTAL MODEL AND SUBJECT DETAILS
- METHOD DETAILS
  - MRI data acquisition
  - Day 1: FMRI neurofeedback and DTI
  - Functional localizer
  - DTI
  - Neurofeedback training
  - Sham group
  - Experimental conditions
  - Day 2: DTI
  - EMG acquisition
  - Neurofeedback questionnaire
- QUANTIFICATION AND STATISTICAL ANALYSIS
  - fMRI preprocessing
  - ROI fMRI analysis of the feedback training
  - Group-level voxel-wise fMRI analysis
  - DTI analysis
  - Correlations between fMRI change and FA change
  - Tractography analysis
  - EMG analysis
  - Questionnaire analysis

SUPPLEMENTAL INFORMATION

Supplemental information can be found online at https://doi.org/10.1016/j.celrep.2021.109890.

ACKNOWLEDGMENTS

This work was supported by the Wellcome Trust (WT090955AIA and 110027/Z/15/Z to H.J.-B.). The research was further supported by Marie Curie Actions (Adaptive Brain Computations network PITN-GA-2008-290011) and by the National Institute for Health Research (NIHR) Oxford Biomedical Research Centre based at Oxford University Hospitals NHS Trust and University of Oxford. The views expressed are those of the author(s) and not necessarily those of the NHS, the NIHR, or the Department of Health. The Wellcome Centre for Integrative Neuroimaging is supported by core funding from the Wellcome Trust (203139/Z/16/Z). The funders had no role in study design, data collection and analysis, decision to publish, or preparation of the manuscript. The authors would like to thank N. Filippini, S. Foxley, and the radiography team for operating the 7T scanner; T. Makin and F. Eippert for assistance with the EMG; S. Sotiropoulos for assistance with the 3D tractography pictures; W. Richardson, T. Makin, and J. O'Shea for the useful comments on the manuscript; and D. Flitney for computing support. The authors thank the editors and anonymous reviewers for their constructive comments and for reviewing this manuscript in this challenging period.

AUTHOR CONTRIBUTIONS

C.S.-B. designed the study and collected, analyzed, and interpreted the data. H.F.N. collected data and assisted with data analysis. Z.-B.S. collected and analyzed data. D.H and K.D. collected data. Y.H. provided assistance with EMG data analysis. J.L.R.A., M.L., and R.G. developed and provided assistance with data collection methods and analysis. H.J.-B. designed the study and supervised the project. C.S.-B. wrote the manuscript and all authors edited it.

DECLARATION OF INTERESTS

R.G. is CEO and M.L. is an employee of Brain Innovation B.V., which makes the Turbo-Brain Voyager software used for the real-time fMRI analyses. The remaining authors declare no competing interests.

Received: June 21, 2021
Revised: August 6, 2021
Accepted: October 5, 2021
Published: October 26, 2021

REFERENCES

Andersson, J.L.R., and Sotiropoulos, S.N. (2016). An integrated approach to correction for off-resonance effects and subject movement in diffusion MR imaging. Neuroimage *125*, 1063–1078.

Andersson, J.L., Skare, S., and Ashburner, J. (2003). How to correct susceptibility distortions in spin-echo echo-planar images: application to diffusion tensor imaging. Neuroimage *20*, 870–888.

Arancibia-Cárcamo, I.L., Ford, M.C., Cossell, L., Ishida, K., Tohyama, K., and Attwell, D. (2017). Node of Ranvier length as a potential regulator of myelinated axon conduction speed. eLife *6*, e23329.

Auer, T., Schweizer, R., and Frahm, J. (2015). Training Efficiency and Transfer Success in an Extended Real-Time Functional MRI Neurofeedback Training of the Somatomotor Cortex of Healthy Subjects. Front. Hum. Neurosci. *9*, 547.

Bacmeister, C.M., Barr, H.J., McClain, C.R., Thornton, M.A., Nettles, D., Welle, C.G., and Hughes, E.G. (2020). Motor learning promotes remyelination via new and surviving oligodendrocytes. Nat. Neurosci. *23*, 819–831.

Baraban, M., Koudelka, S., and Lyons, D.A. (2018). Ca $^{2+}$ activity signatures of myelin sheath formation and growth in vivo. Nat. Neurosci. *21*, 19–23.

Behrens, T.E.J., Berg, H.J., Jbabdi, S., Rushworth, M.F.S., and Woolrich, M.W. (2007). Probabilistic diffusion tractography with multiple fibre orientations: What can we gain? Neuroimage *34*, 144–155.

Blumenfeld-Katzir, T., Pasternak, O., Dagan, M., and Assaf, Y. (2011). Diffusion MRI of structural brain plasticity induced by a learning and memory task. PLoS ONE *6*, e20678.

Bradnam, L.V., Stinear, C.M., Barber, P.A., and Byblow, W.D. (2012). Contralesional hemisphere control of the proximal paretic upper limb following stroke. Cereb. Cortex *22*, 2662–2671.

Chiew, M., LaConte, S.M., and Graham, S.J. (2012). Investigation of fMRI neurofeedback of differential primary motor cortex activity using kinesthetic motor imagery. Neuroimage *61*, 21–31.

Czopka, T., Ffrench-Constant, C., and Lyons, D.A. (2013). Individual oligodendrocytes have only a few hours in which to generate new myelin sheaths in vivo. Dev. Cell *25*, 599–609.

Demerens, C., Stankoff, B., Logak, M., Anglade, P., Allinquant, B., Couraud, F., Zalc, B., and Lubetzki, C. (1996). Induction of myelination in the central nervous system by electrical activity. Proc. Natl. Acad. Sci. USA *93*, 9887–9892.

Devor, A., Tian, P., Nishimura, N., Teng, I.C., Hillman, E.M., Narayanan, S.N., Ulbert, I., Boas, D.A., Kleinfeld, D., and Dale, A.M. (2007). Suppressed neuronal activity and concurrent arteriolar vasoconstriction may explain negative blood oxygenation level-dependent signal. J. Neurosci. *27*, 4452–4459.

Duncan, I.D., Radcliff, A.B., Heidari, M., Kidd, G., August, B.K., and Wierenga, L.A. (2018). The adult oligodendrocyte can participate in remyelination. Proc. Natl. Acad. Sci. USA *115*, E11807–E11816.

Dutta, D.J., Woo, D.H., Lee, P.R., Pajevic, S., Bukalo, O., Huffman, W.C., Wake, H., Basser, P.J., SheikhBahaei, S., Lazarevic, V., et al. (2018). Regulation of myelin structure and conduction velocity by perinodal astrocytes. Proc. Natl. Acad. Sci. USA *115*, 11832–11837.

Ford, M.C., Alexandrova, O., Cossell, L., Stange-Marten, A., Sinclair, J., Kopp-Scheinpflug, C., Pecka, M., Attwell, D., and Grothe, B. (2015). Tuning of Ranvier node and internode properties in myelinated axons to adjust action potential timing. Nat. Commun. *6*, 8073.

Ghaziri, J., Tucholka, A., Larue, V., Blanchette-Sylvestre, M., Reyburn, G., Gilbert, G., Lévesque, J., and Beauregard, M. (2013). Neurofeedback training induces changes in white and gray matter. Clin. EEG Neurosci. *44*, 265–272.

Gibson, E.M., Purger, D., Mount, C.W., Goldstein, A.K., Lin, G.L., Wood, L.S., Inema, I., Miller, S.E., Bieri, G., Zuchero, J.B., et al. (2014). Neuronal activity promotes oligodendrogenesis and adaptive myelination in the mammalian brain. Science *344*, 1252304.

Greve, D.N., and Fischl, B. (2009). Accurate and robust brain image alignment using boundary-based registration. Neuroimage *48*, 63–72.

Haugg, A., Sladky, R., Skouras, S., McDonald, A., Craddock, C., Kirschner, M., Herdener, M., Koush, Y., Papoutsi, M., Keynan, J.N., et al. (2020). Can we predict real-time fMRI neurofeedback learning success from pretraining brain activity?*Hum*. Brain Mapp *41*, 3839–3854.

Hines, J.H., Ravanelli, A.M., Schwindt, R., Scott, E.K., and Appel, B. (2015). Neuronal activity biases axon selection for myelination in vivo. Nat. Neurosci. *18*, 683–689.

Hofstetter, S., Tavor, I., Tzur Moryosef, S., and Assaf, Y. (2013). Short-term learning induces white matter plasticity in the fornix. J. Neurosci. *33*, 12844–12850.

Johansen-Berg, H. (2003). Motor physiology: a brain of two halves. Curr. Biol. *13*, R802–R804.

Johansen-Berg, H. (2007). Functional imaging of stroke recovery: what have we learnt and where do we go from here? Int. J. Stroke *2*, 7–16.

Johansen-Berg, H., Dawes, H., Guy, C., Smith, S.M., Wade, D.T., and Matthews, P.M. (2002a). Correlation between motor improvements and altered fMRI activity after rehabilitative therapy. Brain *125*, 2731–2742.

Johansen-Berg, H., Rushworth, M.F., Bogdanovic, M.D., Kischka, U., Wimalaratna, S., and Matthews, P.M. (2002b). The role of ipsilateral premotor cortex in hand movement after stroke. Proc. Natl. Acad. Sci. USA *99*, 14518–14523.

Logothetis, N.K. (2008). What we can do and what we cannot do with fMRI. Nature *453*, 869–878.

Long, P., and Corfas, G. (2014). Neuroscience. To learn is to myelinate. Science *346*, 298–299.

Marins, T., Rodrigues, E.C., Bortolini, T., Melo, B., Moll, J., and Tovar-Moll, F. (2019). Structural and functional connectivity changes in response to short-term neurofeedback training with motor imagery. Neuroimage *194*, 283–290.

McDonnell, M.N., and Stinear, C.M. (2017). TMS measures of motor cortex function after stroke: A meta-analysis. Brain Stimul. *10*, 721–734.

McKenzie, I.A., Ohayon, D., Li, H., de Faria, J.P., Emery, B., Tohyama, K., and Richardson, W.D. (2014). Motor skill learning requires active central myelination. Science *346*, 318–322.

Mitew, S., Gobius, I., Fenlon, L.R., McDougall, S.J., Hawkes, D., Xing, Y.L., Bujalka, H., Gundlach, A.L., Richards, L.J., Kilpatrick, T.J., et al. (2018). Pharmacogenetic stimulation of neuronal activity increases myelination in an axon-specific manner. Nat. Commun. *9*, 306.

Neyedli, H.F., Sampaio-Baptista, C., Kirkman, M.A., Havard, D., Lührs, M., Ramsden, K., Flitney, D.D., Clare, S., Goebel, R., and Johansen-Berg, H. (2018). Increasing Lateralized Motor Activity in Younger and Older Adults using Real-time fMRI during Executed Movements. Neuroscience *378*, 165–174.

Nichols, T.E., and Holmes, A.P. (2002). Nonparametric permutation tests for functional neuroimaging: a primer with examples. Hum. Brain Mapp. *15*, 1–25.

Pan, S., Mayoral, S.R., Choi, H.S., Chan, J.R., and Kheirbek, M.A. (2020). Preservation of a remote fear memory requires new myelin formation. Nat. Neurosci. *23*, 487–499.

Ramot, M., Grossman, S., Friedman, D., and Malach, R. (2016). Covert neurofeedback without awareness shapes cortical network spontaneous connectivity. Proc. Natl. Acad. Sci. USA *113*, E2413–E2420.

Sampaio-Baptista, C., and Johansen-Berg, H. (2017). White Matter Plasticity in the Adult Brain. Neuron *96*, 1239–1251.

Sampaio-Baptista, C., Khrapitchev, A.A., Foxley, S., Schlagheck, T., Scholz, J., Jbabdi, S., DeLuca, G.C., Miller, K.L., Taylor, A., Thomas, N., et al. (2013). Motor skill learning induces changes in white matter microstructure and myelination. J. Neurosci. *33*, 19499–19503.

Sampaio-Baptista, C., Sanders, Z.B., and Johansen-Berg, H. (2018). Structural Plasticity in Adulthood with Motor Learning and Stroke Rehabilitation. Annu. Rev. Neurosci. *41*, 25–40.

Sampaio-Baptista, C., Vallès, A., Khrapitchev, A.A., Akkermans, G., Winkler, A.M., Foxley, S., Sibson, N.R., Roberts, M., Miller, K., Diamond, M.E., et al. (2020). White matter structure and myelin-related gene expression alterations with experience in adult rats. Prog. Neurobiol. *187*, 101770.

Scholz, J., Klein, M.C., Behrens, T.E., and Johansen-Berg, H. (2009). Training induces changes in white-matter architecture. Nat. Neurosci. *12*, 1370–1371.

Shibata, K., Watanabe, T., Sasaki, Y., and Kawato, M. (2011). Perceptual learning incepted by decoded fMRI neurofeedback without stimulus presentation. Science *334*, 1413–1415.

Sinclair, J.L., Fischl, M.J., Alexandrova, O., Heβ, M., Grothe, B., Leibold, C., and Kopp-Scheinpflug, C. (2017). Sound-Evoked Activity Influences Myelination of Brainstem Axons in the Trapezoid Body. J. Neurosci. *37*, 8239–8255.

Sitaram, R., Ros, T., Stoeckel, L., Haller, S., Scharnowski, F., Lewis-Peacock, J., Weiskopf, N., Blefari, M.L., Rana, M., Oblak, E., et al. (2017). Closed-loop brain training: the science of neurofeedback. Nat. Rev. Neurosci. *18*, 86–100.

CellPress
OPEN ACCESS

Smith, S.M., Jenkinson, M., Woolrich, M.W., Beckmann, C.F., Behrens, T.E., Johansen-Berg, H., Bannister, P.R., De Luca, M., Drobnjak, I., Flitney, D.E., et al. (2004). Advances in functional and structural MR image analysis and implementation as FSL. Neuroimage *23* (*Suppl 1*), S208–S219.

Smith, S.M., Jenkinson, M., Johansen-Berg, H., Rueckert, D., Nichols, T.E., Mackay, C.E., Watkins, K.E., Ciccarelli, O., Cader, M.Z., Matthews, P.M., and Behrens, T.E. (2006). Tract-based spatial statistics: voxelwise analysis of multi-subject diffusion data. Neuroimage *31*, 1487–1505.

Steadman, P.E., Xia, F., Ahmed, M., Mocle, A.J., Penning, A.R.A., Geraghty, A.C., Steenland, H.W., Monje, M., Josselyn, S.A., and Frankland, P.W. (2020). Disruption of Oligodendrogenesis Impairs Memory Consolidation in Adult Mice. Neuron *105*, 150–164.

Stevens, B., Tanner, S., and Fields, R.D. (1998). Control of myelination by specific patterns of neural impulses. J. Neurosci. *18*, 9303–9311.

Stinear, C.M., and Ward, N.S. (2013). How useful is imaging in predicting outcomes in stroke rehabilitation? Int. J. Stroke *8*, 33–37.

Stinear, C.M., Barber, P.A., Smale, P.R., Coxon, J.P., Fleming, M.K., and Byblow, W.D. (2007). Functional potential in chronic stroke patients depends on corticospinal tract integrity. Brain *130*, 170–180.

Taubert, M., Draganski, B., Anwander, A., Müller, K., Horstmann, A., Villringer, A., and Ragert, P. (2010). Dynamic properties of human brain structure: learning-related changes in cortical areas and associated fiber connections. J. Neurosci. *30*, 11670–11677.

Thomas, C., and Baker, C.I. (2013). Teaching an adult brain new tricks: a critical review of evidence for training-dependent structural plasticity in humans. Neuroimage *73*, 225–236.

Uhlirova, H., Kılıç, K., Tian, P., Thunemann, M., Desjardins, M., Saisan, P.A., Sakadžić, S., Ness, T.V., Mateo, C., Cheng, Q., et al. (2016). Cell type specificity of neurovascular coupling in cerebral cortex. eLife *5*, e14315.

Wang, T., Mantini, D., and Gillebert, C.R. (2018). The potential of real-time fMRI neurofeedback for stroke rehabilitation: A systematic review. Cortex *107*, 148–165.

Watkins, T.A., Emery, B., Mulinyawe, S., and Barres, B.A. (2008). Distinct stages of myelination regulated by gamma-secretase and astrocytes in a rapidly myelinating CNS coculture system. Neuron *60*, 555–569.

Woolrich, M.W., Behrens, T.E., Beckmann, C.F., Jenkinson, M., and Smith, S.M. (2004). Multilevel linear modelling for FMRI group analysis using Bayesian inference. Neuroimage *21*, 1732–1747.

Xiao, L., Ohayon, D., McKenzie, I.A., Sinclair-Wilson, A., Wright, J.L., Fudge, A.D., Emery, B., Li, H., and Richardson, W.D. (2016). Rapid production of new oligodendrocytes is required in the earliest stages of motor-skill learning. Nat. Neurosci. *19*, 1210–1217.

Yeung, M.S., Zdunek, S., Bergmann, O., Bernard, S., Salehpour, M., Alkass, K., Perl, S., Tisdale, J., Possnert, G., Brundin, L., et al. (2014). Dynamics of oligodendrocyte generation and myelination in the human brain. Cell *159*, 766–774.

Young, K.D., Siegle, G.J., Zotev, V., Phillips, R., Misaki, M., Yuan, H., Drevets, W.C., and Bodurka, J. (2017). Randomized Clinical Trial of Real-Time fMRI Amygdala Neurofeedback for Major Depressive Disorder: Effects on Symptoms and Autobiographical Memory Recall. Am. J. Psychiatry *174*, 748–755.

STAR★METHODS

KEY RESOURCES TABLE

REAGENT OR RESOURCE	SOURCE	IDENTIFIER
Software and algorithms		
FSL (FMRIB SOFTWARE LIBRARY)	Analysis group, FMRIB,WIN, University of Oxford, UK	https://fsl.fmrib.ox.ac.uk/fsl/fslwiki
Turbo Brain Voyager	Brain Innovation B.V. Oxfordlaan 55 6229 EV Maastricht the Netherlands	https://www.brainvoyager.com/TurboBrainVoyager.html

RESOURCE AVAILABILITY

Lead contact

Further information and requests for resources and reagents should be directed to and will be fulfilled by the lead contact, Cassandra Sampaio Baptista cassandra.sampaiobaptista@Glasgow.ac.uk

Materials availability

This study did not generate new unique reagents.

Data and code availability

This study did not generate new code. Participants did not provide informed consent to share their data. Group level data supporting the study can be made available. Other than the exceptions stipulated above, any additional information required to reanalyze the data reported in this paper is available from the lead contact upon request.

EXPERIMENTAL MODEL AND SUBJECT DETAILS

All participants provided written informed consent in accordance with the University of Oxford ethics committee approval of the protocol (MSD-IDREC-C1-2012-151).

28 right-handed participants (22-38 years old, 15 female) were recruited and scanned in a 7T Siemens scanner 4 times (Figure 1A).

Participants were blind to group assignment: Real Neurofeedback (NF) or Sham NF (more details below). Total number of analyzed scans was 80 (Real NF group n = 10x4 = 40; Sham group n = 10x4 = 40).

Five participants dropped out due to a variety of reasons (e.g back pain or claustrophobia) unrelated to the neurofeedback group/condition. Data were not fully collected due to technical issues (i.e., 7T scanner crashes) in three participants. In more detail, one participant in the Real NF group did not complete the experiment due to back pain and DTI was not acquired in one other participant due to scanner crashes. Four participants in the Sham group did not complete the full study due to back pain or claustrophobia and data were not fully collected in further two Sham participants due to scanner crashes.

The experimenter could not be blinded to group due to limitations of the real-time software. However, all participants received identical instructions and experimental procedures were the same across the two groups with the exception of the source of the feedback presented (see below). Each participant in each group was scanned under two experimental conditions: association and Dissociation. For each condition participants were scanned twice, 24 hours apart. The order of experimental conditions was counterbalanced across participants and conditions were spaced at least 2 weeks apart (mean = 33.8 days, SD = 16.3) (see Figure 1A).

METHOD DETAILS

MRI data acquisition

Imaging was performed on a 7.0T Siemens Magnetom MRI system (Siemens, Erlangen, Germany) with a 32-channel head coil at the FMRIB Centre at the University of Oxford. For each condition, scans were acquired over two days as follows:

Day 1: FMRI neurofeedback and DTI

All FMRI data were acquired with a gradient echo planar image sequence (16 slices, 2 mm axial plane, 2×2 mm^2 in plane resolution, repetition time (TR) = 2000 ms; echo time (TE) = 25 ms; flip angle = 90°). A whole brain echo planar image sequence was acquired for registration purposes (60 slices, 2 mm axial plane, 2×2 mm^2 in plane resolution, TR = 3500 ms; echo time = 25 ms; flip angle = 90° field of view, 220×220mm).

Functional localizer

The functional localizer consisted of eight, 12 s tapping blocks, four blocks for each hand, interspersed with 24 s rest. The participants saw the instructions 'Right Tap', 'Left Tap' and 'Rest' displayed in white on a black background. Participants were told to use each finger in sequence starting with their index finger and moving outward toward the little finger at a rate of approximately 1 Hz, and to repeat the sequence until they saw the rest instruction. The results from the real-time general linear model (GLM) of the localizer scan were used to select two motor ROIs (18 × 18 × 10mm) for each participant, each centered over the peak of activation in both hemispheres.

DTI

After the functional localizer we acquired whole brain diffusion-weighted volumes (64 directions; b-value = 1500 s/mm^2; 80 slices; voxel size 1.5 × 1.5 × 1.5 mm^3; TR = 10 s; TE = 64 ms) including 5 volumes without diffusion weighting (b-value = 0 s/mm^2)), and also a separate dataset without diffusion weighting with opposite phase encoding for correction of susceptibility induced distortions (b-value = 0 s/mm^2, 80 slices; voxel size 1.5 × 1.5 × 1.5 mm^3; repetition time (TR) = 10 s; echo time (TE) = 64 ms). The total acquisition time for DTI was about 10 minutes.

Neurofeedback training

Next, three, ~6 min NF functional scans were acquired with a block design (30 s on, 30 s rest). In five sessions a fourth NF scan was acquired but only the first 3 training runs that were common to all participants were analyzed.

Turbo-BrainVoyager software version 3.2 (Brain Innovation, Maastricht, the Netherlands) was used to calculate the BOLD signal in real time using a whole-brain voxel-wise recursive GLM. The feedback signal was based on the averaged voxel time-course extracted from the localized motor ROIs and the feedback image was updated each TR (2 s). Online motion correction in three dimensions, including translations and rotations were used to correct for head movements during the scan.

A custom-made transmission control protocol (TCP) based network interface plug-in for Turbo-Brain Voyager was used to transmit the preprocessed ROI time-course to Turbo-Feedback, a custom-made software tool, which performed NF signal calculation and displayed the resulting feedback to the participants continuously (every TR).

Participants saw two vertical bars, representing the contralateral and ipsilateral hemispheric activity, with a horizontal line delineating the center point. The equation used to calculate the height of each bar was the following:

$$\text{NF signal} = ([\text{ROI}_{act} - \text{ROI}_{rest}] / \text{ROI}_{rest})$$

$$\text{Bar Height on the display} = ([\text{ROI}_{act} - \text{ROI}_{rest}] / \text{ROI}_{rest})/\text{MaxBarHeight}$$

where ROI_{act} = current BOLD signal in ROI

ROI_{rest} = mean BOLD signal in the ROI during the previous rest blocks.
MaxBarHeight = maximum level of the bar

Positive values were represented above the center point, and negative values below the center point (Figures 1C and 1D). The bar for contralateral (right hemisphere) activation was displayed on the left as this hemisphere should be most active during left hand movement. The bar for ipsilateral (left hemisphere) activation was displayed on the right.

Sham group

Participants in the Sham group were matched to a participant in the NF group and received feedback videos from that participant (rather than their feedback from their own brain activity). This allowed the Sham participants to have a similar experience as the Real NF group. All scans and instructions received by the Sham group were identical to those received by the Real NF group.

Experimental conditions

Two conditions were tested in separate sessions in both the Real NF and the Sham group:

Association condition Participants were instructed to increase the size of both bars

Dissociation condition Participants were instructed to decrease the size of the bar on the right side of the screen, while increasing the bar size on the left side

In this way, the goal of the Association condition was to maximize activation in both left and right S1M1, whereas the goal of the Dissociation condition was to maximize right (contralateral) S1M1 activity and minimize left (ipsilateral) S1M1 activity.

Participants were only told that the bars represented their brain activity. For both conditions participants were asked to perform left hand movements in order to modulate the height of the bars. The participants saw the instructions 'Move Left' or 'Rest' displayed in white on a black background. Participants were allowed to use any left hand movement strategy to accomplish the goal in each condition. Participants were instructed not to move their right hand during NF training and both arms were monitored on-line for

movement using EMG. During the instructions a list of example strategies were read but participants were told they could use any other strategy as long as they did not move their right hand:

> *"Open and close hand, move fingers, make grasping movements, move fingers sequentially or randomly, imagine hand/finger movements, focus on moving hand or non-moving hand, increase rate, force, size, of movement etc."*

Day 2: DTI
24 hours after each NF training session DTI was again acquired with the same parameters as above. For registration purposes, one structural image per subject was acquired during the second session only using a T1 weighted, MPRAGE sequence with 1 × 1 x 1 mm^3 isotropic voxels (TR = 2200 ms; TE = 2.2 ms; flip angle 7°, field of view, 192x192; matrix = 192x192).

EMG acquisition
A Biopac system and AcqKnowledge software (Version 4.2) were used for EMG acquisition during NF sessions. Due to technical difficulties we only acquired a full set of EMG data in 7 participants in the NF group and 9 participants in the Sham group. We used two MRI safe surface electrodes (ConMed corporation, USA) to record from the flexor carpi ulnaris muscle and an additional electrode placed over the elbow olecranon was used as the ground electrode. AcqKnowledge software was used to monitor and record muscle activity during the NF training acquisition with online MRI artifact and line noise correction.

Neurofeedback questionnaire
Following each NF training session participants completed a questionnaire outside the scanner (Table S1). Participants reported on a scale of 1-5 how much control they felt they had over the bar. Then a number of strategies for controlling the FB were presented and participants were asked to report if they used the strategy and, if so, how successful they thought the strategy was on a scale of 1-5.

QUANTIFICATION AND STATISTICAL ANALYSIS

Additional details regarding statistical analysis are provided in results section and figure legends

fMRI preprocessing
BOLD fMRI data for each subject were analyzed with FMRIB's expert analysis tool (FEAT, version 5.98) from the FMRIB software library version 5.0 (https://www.fmrib.ox.ac.uk/fsl). Pre-processing of the images included motion correction with FMRIB's Linear Image Registration Tool (MCFLRIT), brain extraction with BET, spatial smoothing using a Gaussian kernel of 5 mm FWHM, and high-pass temporal filtering of 150 s.

Functional data were first aligned to the whole brain scan and then to the subject's structural image with linear registration (FMRIB's Linear Image Registration Tool, FLIRT), and then optimized using Boundary-Based Registration (Greve and Fischl, 2009). For structural images we used the anatomical processing script (fsl_anat) to robustly correct the bias-field and register the images to standard MNI space. The resulting warp fields were then applied to the functional images.

We used a voxel-based general linear model (GLM), as implemented in FEAT. For each NF training run, the block design paradigm (30 s hand movement plus NF and 30 s rest) convolved with a gamma function, along with its temporal derivative, was used to model the activation time course.

ROI fMRI analysis of the feedback training
After first-level Feat analysis, the tool featquery was used to extract the percentage signal change of the defined motor ROIs. Mixed design ANOVA or Repeated-Measures ANOVA (SPSS version 25) were used when appropriate to test for main effects of group, condition (Association, Dissociation), NF run (1, 2, 3) and interaction effects between these variables. The significance threshold used was $p < 0.05$.

Group-level voxel-wise fMRI analysis
To test for main effects of condition we used a within-subject fixed-effects second-level analysis to calculate the average activation for the contrast of movement versus rest across the three NF scans per participant. The resulting maps were then fed into group level analysis using FMRIB's Local Analysis of Mixed Effects (Woolrich et al., 2004).

We tested for differences between Association and Dissociation conditions with a paired t test.

Z statistic images were thresholded using clusters determined by $Z > 3.1$ and a family-wise-error-corrected cluster significance threshold of $p < 0.05$ was applied to the suprathreshold clusters.

DTI analysis
DTI data were analyzed with FMRIB's Diffusion Toolbox (FDT). Two sets of volumes without diffusion-weighting were collected, with reversed phase-encode blips (i.e., one set with anterior-posterior encoding and one with posterior-anterior), resulting in pairs of

images with distortions going in opposite directions. From these image pairs the susceptibility-induced off-resonance field was estimated using the ‘‘topup’’ tool, with a method similar to that described in (Andersson et al., 2003) as implemented in FSL (Smith et al., 2004). All data were then corrected for susceptibility induced distortions, including and for eddy current distortions and head movements with the FSL’s eddy tool (Andersson and Sotiropoulos, 2016).

A diffusion tensor model was then fit to the data at each voxel using dtifit and voxel-wise maps of fractional anisotropy (FA), mean diffusivity (MD), radial and axial diffusivity were estimated for each subject and each time point. These maps were then analyzed using Tract Based Spatial Statistics (TBSS) (Smith et al., 2006). We performed unbiased registration by registering the maps to the study specific template.

A mixed-design ANOVA is not accommodated by the general linear model (GLM) as implemented in the FSL tool Randomize (https://fsl.fmrib.ox.ac.uk/fsl/fslwiki/Randomise). As such to be able to test for group differences we have first computed the FA change (post-pre) maps for each condition and each participant. We then calculated the difference between conditions for each participant (Dissociation FA change – Association FA change = condition difference). These maps were then compared between groups (Real NF versus Sham) with an unpaired t test. This allows us to test whether differences in FA change between conditions were greater in magnitude in the Real NF group compared to the Sham group. Gender was used as a covariate (2/8 males/females in Real NF group and 4/6 males/females in the Sham group).

We tested for group differences with an unpaired t test by feeding these difference maps into Randomize for permutation-based non-parametric testing of whole-skeleton FA. Clusters were formed at $t > 1.7$ and tested for significance at $p < 0.05$, corrected for multiple comparisons across space (Nichols and Holmes, 2002).

Correlations between fMRI change and FA change

We tested whether subjects who showed the most effective FMRI modulation with NF also had the greatest microstructural change in white matter. To do so, we first calculated change in the fMRI activity (Run 3 – Run 1) for the iS1M1 ROI for each condition. Rather than consider both conditions for each participant, we selected for each participant the condition in which they performed best. By considering only one condition per participant we could also ensure independence of data points for correlation calculation. Best performance was defined as highest activity change in the instructed direction. 40% of the participants responded best to the Association condition and 60% to the Dissociation condition, 50% of the participants performed best in the first session regardless of condition (Table S3). We tested for correlations between this iS1M1 fMRI change and the corresponding FA change (Post24hrs-Baseline) with Spearman’s Rho ($p < 0.05$, 2-tail) (SPSS version 25) (Figure S3A).

Tractography analysis

We used tractography to identify the probabilistic connectivity map of the significant corpus callosum FA cluster (i.e., cluster shown in Figure 3A). First, for each participant, BEDPOSTX was used to automatically determine the number of estimated fiber populations per brain voxel and to fit estimates of principle diffusion direction for each population (Behrens et al., 2007). Then PROBTRACKX (5000 samples, 0.5 mm step length, 2000 steps, 0.2 curvature threshold) was used to follow these estimates in order to generate a probabilistic connectivity distribution, using the FA cluster as a seed. The resulting individual participant probabilistic connectivity maps were thresholded at 100. We then created two maps to illustrate the connectivity of the significant FA cluster. To create the mean probability map the individual maps were overlapped across participants and the mean was extracted (Figure S2B). To represent the tracts common to the population, the maps were binarized, added together and color-coded (Figure S2C).

EMG analysis

EMG data were band pass filtered offline from 20 Hz to 200 Hz, full-wave rectified and converted to root mean square (RMS) using a 50 ms window period. For statistical comparison, response-locked RMS-EMG activity was averaged from 0 to 30 s for each movement block, after subtracting the 3 s before each movement onset as baseline. We used a Mixed Design ANOVA (SPSS version 25) to test for effects of group, condition, run and hand.

Questionnaire analysis

A Wilcoxon signed-rank test was conducted to compare how much control the participants felt they had over the NF between conditions within group (Question A, Table S1). A Mann-Whitney U test was used to compare how much control the participants felt they had over the NF between groups (Question A, Table S1). A Mann-Whitney U test was used to test if there were differences between groups in how successful the strategies were perceived to be (Question B, Table S1).

Cell Reports, Volume *37*

Supplemental information

fMRI neurofeedback in the motor system elicits bidirectional changes in activity and in white matter structure in the adult human brain

Cassandra Sampaio-Baptista, Heather F. Neyedli, Zeena-Britt Sanders, Kata Diosi, David Havard, YunYing Huang, Jesper L.R. Andersson, Michael Lühr, Rainer Goebel, and Heidi Johansen-Berg

Supplementary Figures and Results

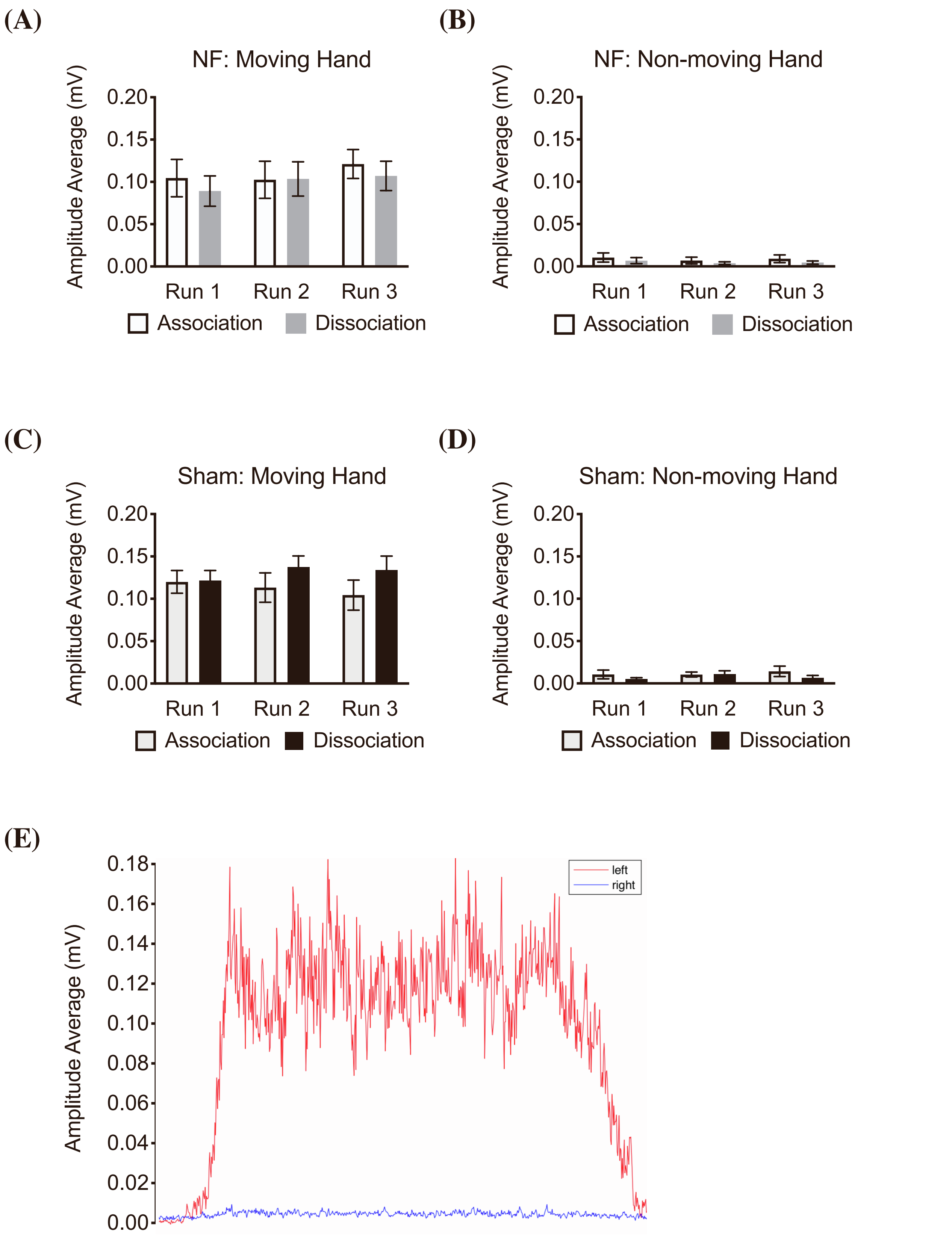

Supplementary Figure 1. EMG results. Related to Figure 2. Mixed Design ANOVA that included group, condition, run and hand as factors revealed an effect of hand, with the average amplitude EMG of the moving (left) hand significantly higher than the non-moving

(right) hand (Fig S1A, B) ($F_{(1,14)}$ = 182.472, $p < 0.001$). There was no effect of group ($F_{(1,14)}$ = 0.522, $p = 0.482$), condition ($F_{(1,14)}$ = 0.006, $p = 0.938$), or run (($F_{(2,28)}$ = 1.508, $p = 0.239$) nor any interaction effects (all $p > 0.05$). (A) Average amplitude (mV) of the left hand for the NF group. (B) Average amplitude of the right hand for the NF group. (C) Average amplitude (mV) of the left hand for the Sham group. (D) Average amplitude of the right hand for the Sham group (E) Example from one participant of the average activation for each hand. Error bars represent SEM.

Questionnaire
A. How much control over the blue bar did you feel you had? (1-not in control, 5-full in control)
B. Which strategies did you use? rate on a scale of 1 to 5 how successful the strategy was (1 – unsuccessful strategy; 5 – successful strategy):
1. Focusing more on the moving hand
2. Focusing less on the non-moving hand
3. Physically relaxing the non-moving hand
4. Increasing the rate of movement
5. Increasing the force of the movement
6. Increasing the size of the movement
7. Tapping the fingers in a fixed sequence
8. Tapping the fingers in a random sequence
9. Opening and closing the hand
10. Making grasping movements
11. Imagining bilateral movements (while keeping the right hand still)
12. - Other strategies (score)

Supplementary Table 1. Debriefing Questionnaire. Related to Figure 2. Participants filled in a questionnaire after the neurofeedback session.

Questionnaire								
A. How much control over the blue bar did you feel you had? (1-not in control, 5-full in control)	NF Group				Sham Group			
	Association		Dissociation		Association		Dissociation	
	Mean	SD	Mean	SD	Mean	SD	Mean	SD
	2.63	1.2	3.1	0.98	2.70	0.59	2.95	0.64

Supplementary Table 2. Debriefing Questionnaire Results. Related to Figure 2. There were no significant differences between sham and NF groups in response to the question “how much control over the blue bar did you feel you had?” for the Association condition (Mann-Whitney U test; Z = 0.327, p=0.756) or the Dissociation condition (Mann-Whitney U test; Z = -1.081, p=0.314). Within group, there were no significant differences in response to this question between experimental conditions (Wilcoxon signed-rank test; real NF group: Z = -1.294, p=0.196; Sham group: Z = -0.991, p=0.322). With regards to participants’ rankings on how useful they found the strategies they tried (Supplementary Table 1, Question B), there was no difference in the rankings between groups for the Association condition (Mann-Whitney U test; Z = 0.986, p = 0.349) or the Dissociation condition (Mann-Whitney U test; Z = 0.458, p = 0.654). Overall, participants of both groups perceived similar degrees of control and considered that the strategies were similarly successful.

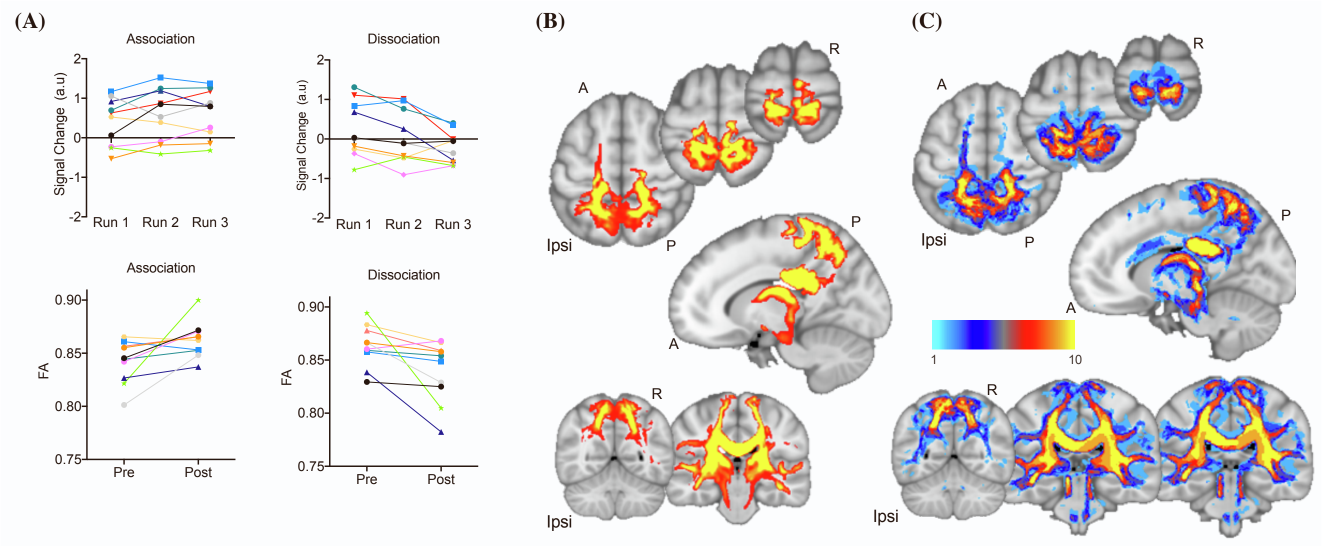

Supplementary Figure 2. Related to Figure 2 and 3. Individual data and tractography results (A) Plots showing colour-coded individual data over time for the Real NF group. Top: Ipsilateral S1M1 activity during fMRI NF for each condition over the 3 runs. Bottom: Pre and Post average FA values for each condition of the significant cluster represented in Figure 3A of the main manuscript. (B-C) The significant FA cluster connects to sensorimotor and posterior parietal areas. (B) Mean connectivity map (red-yellow) of all participants of the FA cluster. Yellow areas depict higher connectivity probability (threshold > 100). (C) Population connectivity map showing the overlap between participants (Light blue represents 1 participant – Yellow represents tracts common to the 10 participants). Tractography map is overlaid on the MNI template. Ipsi – Ipsilateral Hemisphere, A-Anterior, P- Posterior, R - Right.

Participant	Session	Condition
1	1	Association
2	2	Dissociation
3	2	Dissociation
4	1	Dissociation
5	1	Association
6	2	Association
7	1	Dissociation
8	2	Dissociation
9	1	Dissociation
10	2	Association

Supplementary Table 3. Related to Figure 3. Session and condition of best performance for each participant. We expected that changes in white matter structure would reflect successful modulation of activity with NF. For each participant we therefore identified which of the two NF conditions they performed best and computed correlations (see Supplementary Figure 3. A and B).

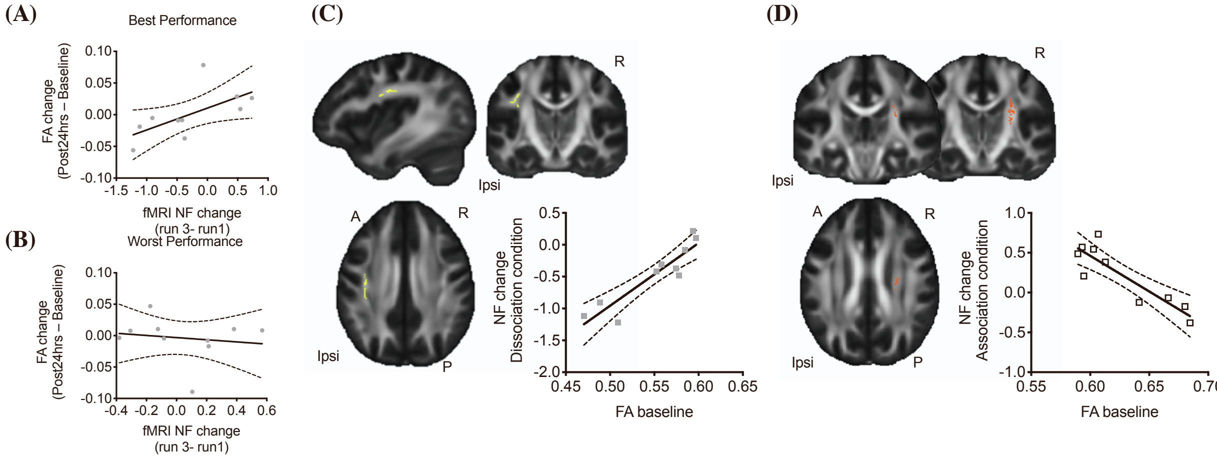

Supplementary Figure 3. Related to Figure 3. Preliminary findings: Correlations between Neurofeedback performance and changes in white matter structure and Baseline FA correlates with NF performance. (A) Significant correlation between the fMRI activity change (run 3 – run 1) and change in FA following the training session with the best performance (Post24hrs – Baseline) (n=10, Spearman's Rho = 0.72, p = 0.02, 2-tail; Supplementary Figure 3A). (B) No such correlation was found for the worse NF session (n=10, Spearman's Rho = - 0.16, p = 0.66, 2-tail; Supplementary Figure 3B). These correlations were significantly different from each other (test of the difference between two dependent correlations; z = 2.058, p = 0.039, 2-tail. Due to the small sample these results should be considered preliminary findings but suggest that following effective NF training, changes in structure are potentially related to how effectively the participant modulated iS1M1 activity. (C) Within the real NF group we tested whether baseline measures of FA correlated with change in fMRI (run 3 – run 1) for each condition (n=10). Non-parametric permutations voxel-wise analysis of the whole skeleton revealed a significant positive correlation ($p < 0.05$, corrected) between baseline FA and NF fMRI change for the Dissociation condition in the ipsilateral (left) superior longitudinal fasciculus (SLF) (represented in yellow). (D) A trend towards a significant negative correlation (p = 0.06, corrected) (represented in red) between baseline FA and NF change was found in the contralateral (right) corticospinal tract for the Association condition. Plots in (C) and (D) are shown for visualization of range of values and not for inference. Significant clusters are superimposed on the FMRIB FA template. Ipsi – Ipsilateral Hemisphere, A-Anterior, P-Posterior, R – Right. Dotted lines represent SEM.
